# 

**Published:** 1897-12

**Authors:** 


ESTABLISHED 1855. SUBSCRIPTION, $2.00.

### ATLANTA MEDICAL AND SURGICAL JOURNAL.

VOLUME XIV NEW SERIES. ATLANTA, GA., DECEMBER, 1897. No. 10.

